# Advancements in predicting and modeling rare event outcomes for enhanced decision-making

**DOI:** 10.1186/s12874-023-02060-x

**Published:** 2023-10-18

**Authors:** Cindy Feng, Longhai Li, Chang Xu

**Affiliations:** 1 Department of Community Health and Epidemiology, Faculty of Medicine, Dalhousie University, 5790 University Ave., Halifax, NS B3H 1V7 Canada; 2 School of Public Health, University of Saskatchewan, 104 Clinic Place, Saskatoon, SK S7N2Z4 Canada; 3 Department of Mathematics and Statistics, University of Saskatchewan, 106 Wiggins Road, Saskatoon, SK S7N5E6 Canada; 4 Key Laboratory for Population Health Across-Life Cycle, Anhui Medical University, Ministry of Education, Anhui, China; 5 School of Public Health, Anhui Medical University, Anhui, China

## Abstract

Predicting rare events is a challenging task due to limited data and imbalanced datasets. This special issue explores methodological advancements in prediction and modeling for rare events. The research showcased in this issue aims to provide valuable insights and strategies to enhance the accuracy of rare event prediction and modeling.

**Editorial:**

Predicting rare event outcomes is of utmost importance across various domains, as it enables early identification of high-risk individuals and facilitates targeted interventions for prevention or mitigation. It is worth noting that rarity refers to events occurring infrequently or having a significantly low prevalence within a specific population, geographic area, or time frame under consideration. Accurate prediction of such events profoundly impacts population health and medication safety. Furthermore, improved prediction models contribute to more efficient and effective clinical trial designs, expediting the development of new treatments for rare diseases. For example, during the initial phase of emerging infectious diseases like COVID-19, the number of cases is typically limited, and the virus can be considered a rare event in the sense that it has not yet spread widely in the population. Similarly, understanding the impact of climate change on emerging diseases, such as heat-related illnesses or emerging vector-borne diseases, during their initial phases is crucial for designing preventive measures and mitigating risks. Additionally, rare events, such as certain types of cancer and medical conditions like neonatal diabetes mellitus, require accurate prediction for early diagnosis and treatment. Nevertheless, predicting rare events poses notable challenges due to limited data availability and imbalanced datasets. These events often occur infrequently and are characterized by limited understanding, which poses challenges in developing accurate prediction models [1, 2]. Furthermore, imbalanced datasets containing rare events alongside numerous non-events introduce biases that favour non-event predictions, leading to poor performance in rare event prediction [1, 2].

In recent years, the field of rare event prediction has witnessed the emergence of several methods aimed at addressing these challenges and developing accurate prediction models. Logistic regression, a widely used method, offers the advantage of simultaneously controlling for multiple confounders. However, it can be problematic if the number of variables exceeds the number of events, potentially yielding unstable estimates, which is known as “sparse data bias” [2, 3]. To enhance traditional statistical models like logistic regression, advanced techniques such as penalized regression and ensemble learning have been employed [4–7]. These advancements enable better handling of the complexity and heterogeneity often encountered in rare event data. Additionally, zero-inflated models have proven to be a suitable approach [8], accounting for excessive zeros and treating them as a separate process when rare events occur infrequently or exhibit excessive zeros. Accounting for the correlation among data, such as spatial or temporal dependencies, may enhance the predictive performance of models for rare event outcomes by incorporating relevant contextual information and capturing the underlying relationships within the data. These methods contribute to the development of more accurate prediction models for rare event data. Other machine learning techniques, including decision trees, random forests, and support vector machines, have demonstrated superior performance compared to clinically used risk calculators when applied to real-world patient data, such as claims or electronic health records [9]. Additionally, deep learning techniques, such as neural networks, have gained remarkable achievements in diverse domains [1]. These methods effectively address imbalanced datasets and yield promising outcomes, although they require substantial amounts of training data and necessitate caution against overfitting in limited data scenarios.

Despite the significant progress made in predicting rare event data, several gaps persist in the existing literature. One major gap pertains to the lack of standardized evaluation metrics for assessing the performance of rare event prediction models. Clear and consistent evaluation metrics specifically designed for imbalanced datasets and rare event prediction models are crucial for meaningful comparisons between different models. Without such metrics, it becomes challenging to evaluate and compare the effectiveness of various rare event prediction approaches. Another significant challenge in predicting rare events is the interpretability of prediction models, especially with complex techniques like deep learning, often seen as black-box models. These models are hard to grasp in terms of decision-making processes, limiting their use in critical areas like healthcare. To overcome this challenge, more research is required to create interpretable models or methods that help understand predictions made by existing models.

Determining appropriate sample sizes for training rare event prediction models is also a challenging task. Traditional sample size calculations that assume equal prevalence between event and non-event groups may not be suitable for rare event modeling. The concept of the “events per variable” (EPV) ratio has been proposed as a guideline, but it may not accurately account for the complexity and heterogeneity of rare event data [10]. More accurate methods are required to determine appropriate sample sizes specifically tailored to the challenges posed by rare event modeling. Furthermore, research aiming to predict rare events with limited data is critical due to under-diagnosis and challenges in understanding these events. Developing methods that learn from limited data while ensuring accurate predictions is essential to address this challenge.

This special issue is dedicated to showcasing the latest research on prediction methods for rare diseases and outcomes. We invite contributions that highlight advancements in statistical modeling, machine learning, and relevant fields, with a focus on exploring implications for clinical practice and research. Our goal is to address existing gaps in the literature, inspire progress in predicting rare event outcomes, and present cutting-edge research.

We encourage submissions in multiple key areas. Firstly, we invite papers that focus on creating and validating prediction models tailored to rare diseases or outcomes. Authors are encouraged to share novel methodologies in this field. Additionally, we welcome discussions regarding the difficulties and advancements in modeling rare event data, emphasizing the challenges of working with limited data and emerging techniques to address them. Furthermore, we are interested in contributions that showcase the practical applications of prediction models in diagnosing diseases that have not been previously identified, illustrating how these models can be effectively used in real-world healthcare settings. Lastly, we seek studies that conduct comparative analyses of various prediction methods for rare diseases or outcomes, offering valuable insights into the pros and cons of different approaches. We welcome contributions that advance the field of rare event prediction while also filling gaps in existing literature.

## Data Availability

Not applicable.

## List of Abbreviations

COVID-19: Coronavirus Disease 2019
EPV: Events per variable
